# Risk of new-onset glaucoma in people with carpal tunnel syndrome: a global-federated, multicenter retrospective cohort study

**DOI:** 10.7150/ijms.122874

**Published:** 2026-01-14

**Authors:** Yu-Jung Su, Shuo-Yan Gau, Yow-Ling Shiue

**Affiliations:** 1 Orthopedics Department, Chi-Mei Medical Center, Tainan, Taiwan.; 2 Institute of Biomedical Sciences, National Sun Yat-sen University, Kaohsiung, Taiwan.; 3 Department of Medical Education, Ditmanson Medical Foundation Chia-Yi Christian Hospital, Chiayi City, Taiwan.; 4 Institute of Allergology, Charité - Universitätsmedizin Berlin, Corporate Member of Freie Universität Berlin and Humboldt-Universität zu Berlin, Berlin, Germany.; 5 Department and Graduate Institute of Business Administration, National Taiwan University, Taipei, Taiwan.; 6 Institute of Biomedical Sciences, Institute of Precision Medicine, College of Medicine, National Sun Yat-sen University, Kaohsiung, Taiwan.

**Keywords:** carpal tunnel syndrome, glaucoma, real-world study, epidemiology

## Abstract

**Background:** Carpal tunnel syndrome (CTS) is a common entrapment neuropathy involving chronic inflammation, while glaucoma is an optic neuropathy linked to neuroinflammation and vascular insufficiency. Shared pathogenic mechanisms have been hypothesized, but large-scale epidemiologic evidence is lacking. This study aims to evaluate whether CTS patients present an elevated risk of glaucoma compared to CTS-free individuals.

**Materials and Methods:** We conducted a retrospective cohort study using the TriNetX global research network. Adults diagnosed with CTS were matched 1:1 with CTS-free controls based on demographics, comorbidities, and healthcare utilization. The primary outcome was new-onset glaucoma, with subtypes assessed separately. Cox proportional hazard models were used to estimate hazard ratios (HRs) with 95% confidence intervals (CIs). Sensitivity analyses included alternative matching algorithms, washout periods, and comparisons with autoimmune musculoskeletal diseases.

**Results:** After matching (n = 733,997 per group), CTS was associated with an increased risk of glaucoma (HR = 1.57, 95% CI: 1.52-1.62). Risks were elevated across glaucoma subtypes, including open-angle glaucoma (HR = 1.55, 95% CI: 1.44-1.66) and angle-closure glaucoma (HR = 1.67, 95% CI: 1.38-2.02). Sensitivity analyses confirmed the robustness of the association across multiple models. When compared to patients with autoimmune musculoskeletal diseases, CTS patients had a higher risk of glaucoma than those with rheumatoid arthritis (HR = 1.73, 95% CI: 1.60-1.87) or ankylosing spondylitis (HR = 1.36, 95% CI: 1.20-1.53).

**Conclusion:** Carpal tunnel syndrome is associated with a significantly increased risk of glaucoma. These findings support the involvement of shared inflammatory or vascular mechanisms and highlight the growing concern about ocular comorbidities in patients with CTS.

## Introduction

Carpal tunnel syndrome (CTS) is a common peripheral entrapment neuropathy caused by compression of the median nerve. Clinically, CTS presents with pain numbness and can lead to grip weakness or thenar muscle atrophy in advanced cases^1^. It is the most frequent entrapment neuropathy in adults, affecting 3-6% of the general population^2^. The clinical burden of CTS is substantial, extending beyond personal discomfort and functional impairment, and contributes to work disability. Risk factors and associated conditions have been identified, including occupational overuse, obesity, and inflammatory disorders^3^. Management ranges from conservative measures, such as wrist splinting or corticosteroid injections, for mild cases to surgical decompression for moderate to severe cases^1^.

Glaucoma is a heterogeneous group of optic neuropathies characterized by progressive degeneration of retinal ganglion cells and their axons, typically associated with intraocular pressure (IOP)-related damage to the optic nerve^4^. Clinically, glaucoma can lead to irreversible vision loss, classically affecting peripheral vision first before advancing centrally. Epidemiologically, glaucoma is one of the most significant causes of vision loss worldwide, which is a leading cause of irreversible blindness, affecting an estimated more than 4 million individuals in the United States^5^. Risk factors for glaucoma include advanced age, a family history of the condition, and elevated intraocular pressure, all of which increase the likelihood of developing the disease^6^. Additionally, chronic inflammatory or autoimmune conditions, such as ankylosing spondylitis and rheumatoid arthritis, are also considered potential risk factors for glaucoma^6^-^9^.

Emerging evidence indicates that there may be systemic or mechanistic overlaps between the two conditions, such as chronic low-grade inflammation, immune-mediated tissue damage, or microvascular dysfunction affecting neural tissues^10^, ^11^. Despite these theoretical connections, the association between CTS and glaucoma has not been directly examined in large patient cohorts until now. Identifying a link would fill a gap in our understanding of glaucoma's systemic risk profile and suggest new prevention avenues. Therefore, we aimed to evaluate whether CTS patients present an elevated risk of glaucoma compared to CTS-free individuals using a global multicenter electronic health record database to address this knowledge gap.

## Materials and Methods

### Data Source and Study Framework

This study employed a retrospective cohort approach using TriNetX global health research network data. TriNetX compiles continuously refreshed, anonymized electronic medical records from numerous health institutions and has been widely used in prior studies focused on healthcare outcomes and economics^12^, ^13^. For this analysis, we utilized data from the Global Collaborative Network segment, comprising records from 147 healthcare providers across North and South America, Europe, and Asia, collectively encompassing over 144 million patient records. The research adhered to ethical standards outlined in the Declaration of Helsinki and was approved with an exemption from informed consent by the Chi Mei Medical Center's IRB (Approval No.: 11312-E01).

### Participant Criteria

Participants were identified using administrative codes corresponding to diagnoses, treatments, and medications (listed in **Table S1**), spanning data collected between 2005 and 2023. The CTS cohort included individuals with at least two clinical visits and a diagnosis record of CTS. At the same time, the comparison group comprised those receiving general health checkups with no CTS history. Exclusion criteria included individuals under 18 years old, those with cancer histories, deceased patients, and anyone diagnosed with glaucoma on or before the study's reference date. We applied 1:1 propensity score matching to balance baseline characteristics between groups.

### Study Model and Variable Control

To mitigate confounding, we conducted a matched cohort analysis. Propensity scores were calculated based on variables such as age at cohort entry, gender, race, BMI, hospitalization records, substance use-related disorders, socioeconomic vulnerabilities, and key comorbid conditions, including hypertension and rheumatoid arthritis. The primary endpoint was a new diagnosis of glaucoma, with follow-up ending at the first occurrence of the outcome event. In this study, the outcome “glaucoma” was defined according to ICD-10-CM codes H40-H42. This definition includes both glaucoma and glaucoma-related conditions such as glaucoma suspect, preglaucoma, open-angle borderline findings, anatomical narrow angle, and ocular hypertension.

### Subgroup and Sensitivity Analyses

To validate the robustness of our findings, we implemented alternative matching algorithms and applied washout intervals to reduce bias from reverse causation. Details of these models can be found in **Table S2**. Additional stratified analyses were conducted based on demographic variables like age group, gender, and ethnicity.

### Statistical Methods

Analyses were executed using TriNetX's embedded statistical tools. Hazard ratios (HRs) and 95% confidence intervals (CIs) were calculated to estimate the relative risk of glaucoma between groups. We assessed covariate balance using the Standardized Mean Difference (SMD), with values under 0.1 indicating proper matching with no significant difference between groups.

## Results

### Baseline Characteristics

**Figure 1:**
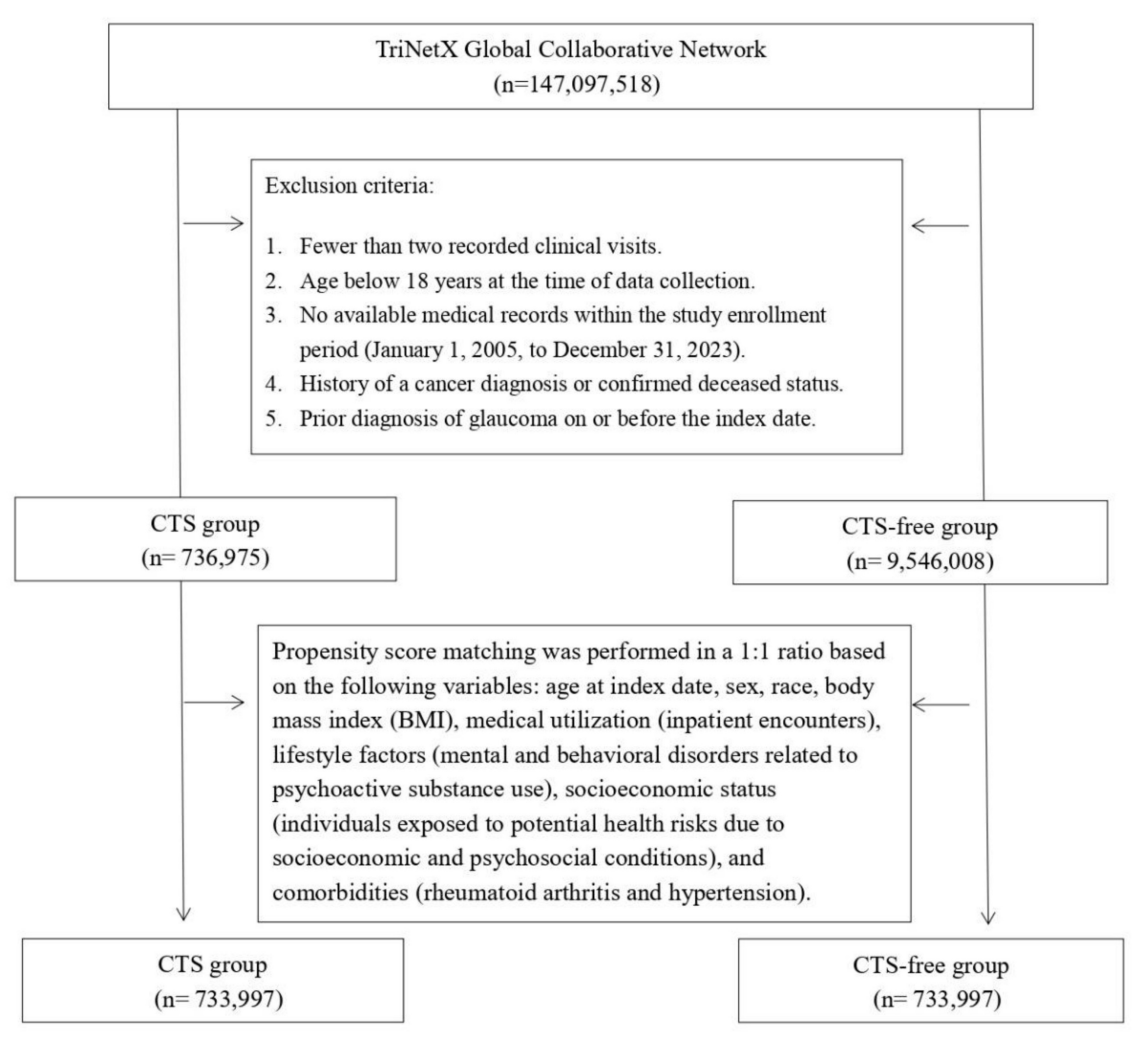
Flowchart of the patient selection process.

Before matching, CTS group enrolled 736,975 patients, and the CTS-free group enrolled 9,546,008 patients (**Figure 1**). **Table 1** presents baseline characteristics of CTS and CTS-free cohorts before and after 1:1 propensity score matching. Before matching, CTS patients were older than controls (mean age 50.9 ± 15.7 vs. 38.0 ± 20.1 years; SMD = 0.71) and included more females (63.3% vs. 52.9%; SMD = 0.21). Racial distributions differed modestly (e.g., Asian: 4.0% vs. 4.9%; SMD = 0.04). CTS patients had higher rates of socioeconomic hazards (1.9% vs. 1.2%; SMD = 0.05), psychoactive substance use disorders (11.9% vs. 5.1%; SMD = 0.25), inpatient encounters (24.4% vs. 14.1%; SMD = 0.26), and BMI ≥ 25 kg/m² (40.4% vs. 22.3%; SMD = 0.40). Comorbidities including hypertension (24.0% vs. 11.9%; SMD = 0.32), hyperlipidemia (14.5% vs. 6.7%; SMD = 0.25), diabetes (11.1% vs. 4.6%; SMD = 0.24), and vitamin D deficiency (7.7% vs. 3.3%; SMD = 0.19) were also more prevalent in CTS group. After matching (n = 733,997 per group), most covariates achieved SMDs ≤ 0.10, indicating a proper balance.

### Glaucoma Risk and Subtypes

**Figure 2:**
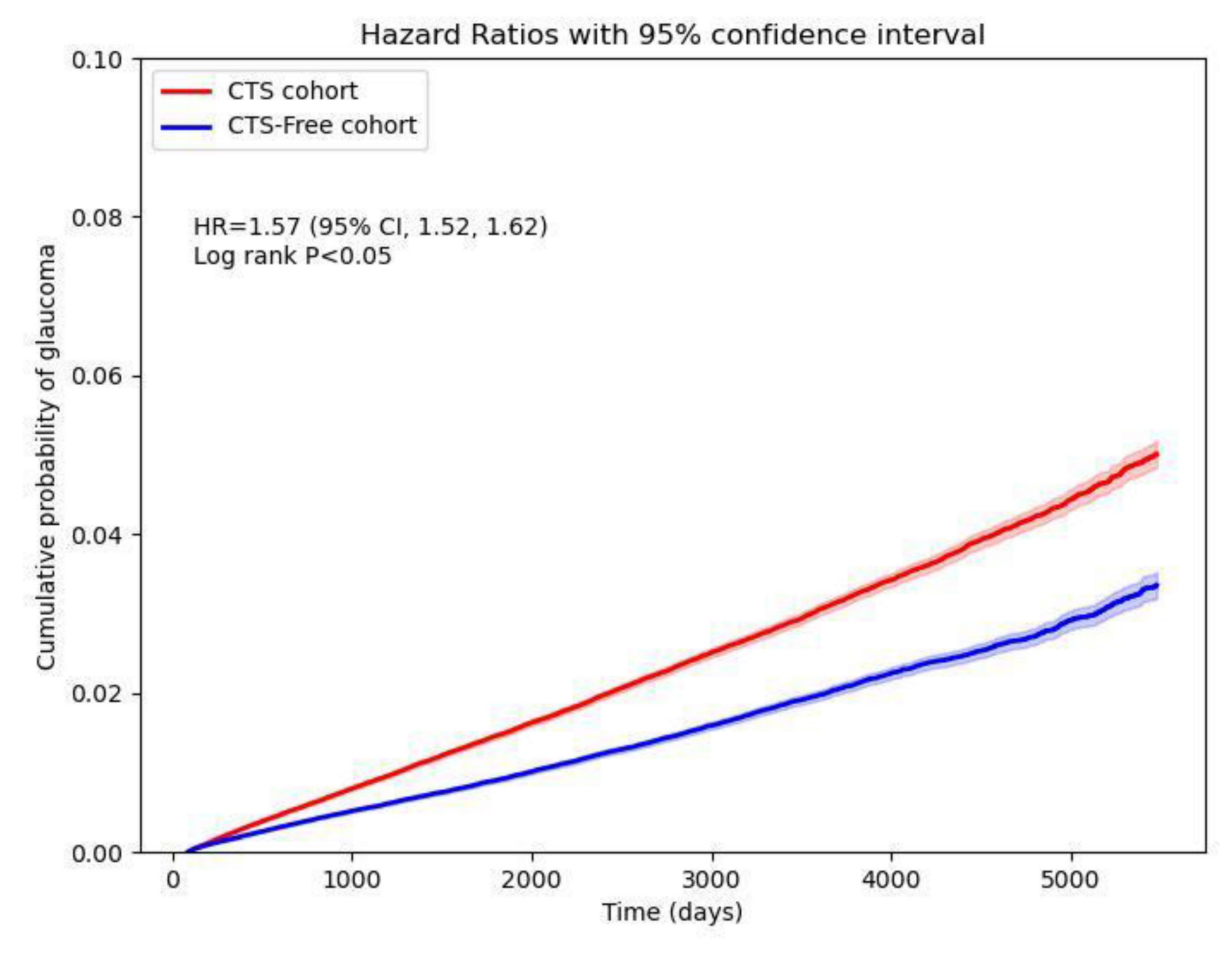
Cumulative probability curve of glaucoma risk in CTS and CTS-free cohorts.

**Figure 3:**
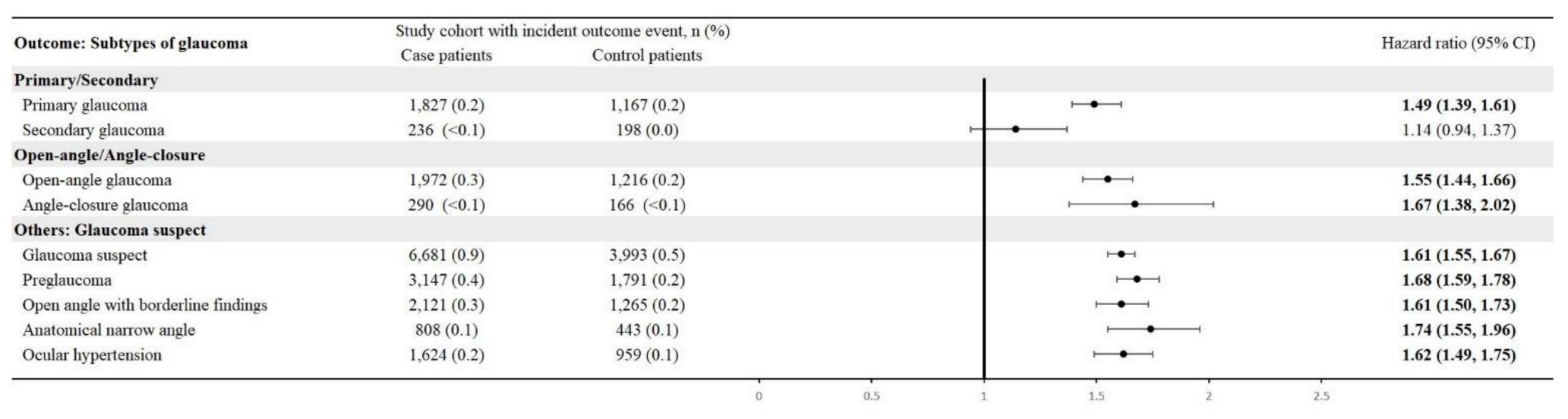
Risk of glaucoma subtypes.

CTS patients were associated with a high risk of developing new-onset glaucoma when compared with CTS-free people, with a hazard ratio of 1.57 (95% CI, 1.52-1.62). **Figure 2** shows the cumulative probability of glaucoma in CTS and CTS-free groups. Primary glaucoma was diagnosed in CTS patients with an HR of 1.49 (95% CI: 1.39-1.61), whereas secondary glaucoma showed an HR of 1.14 (95% CI: 0.94-1.37). Among open-angle variants, open-angle glaucoma carried an HR of 1.55 (95% CI: 1.44-1.66) and anatomical narrow-angle an HR of 1.74 (95% CI: 1.55-1.96). Angle-closure glaucoma had an HR of 1.67 (95% CI: 1.38-2.02). For early or borderline conditions, the glaucoma suspect had an HR of 1.61 (95% CI: 1.55-1.67), preglaucoma 1.68 (95% CI: 1.59-1.78), open-angle borderline findings of 1.61 (95% CI: 1.50-1.73), and ocular hypertension 1.62 (95% CI: 1.49-1.75) (**Figure 3**).

### Sensitivity Analysis

**Figure 4:**
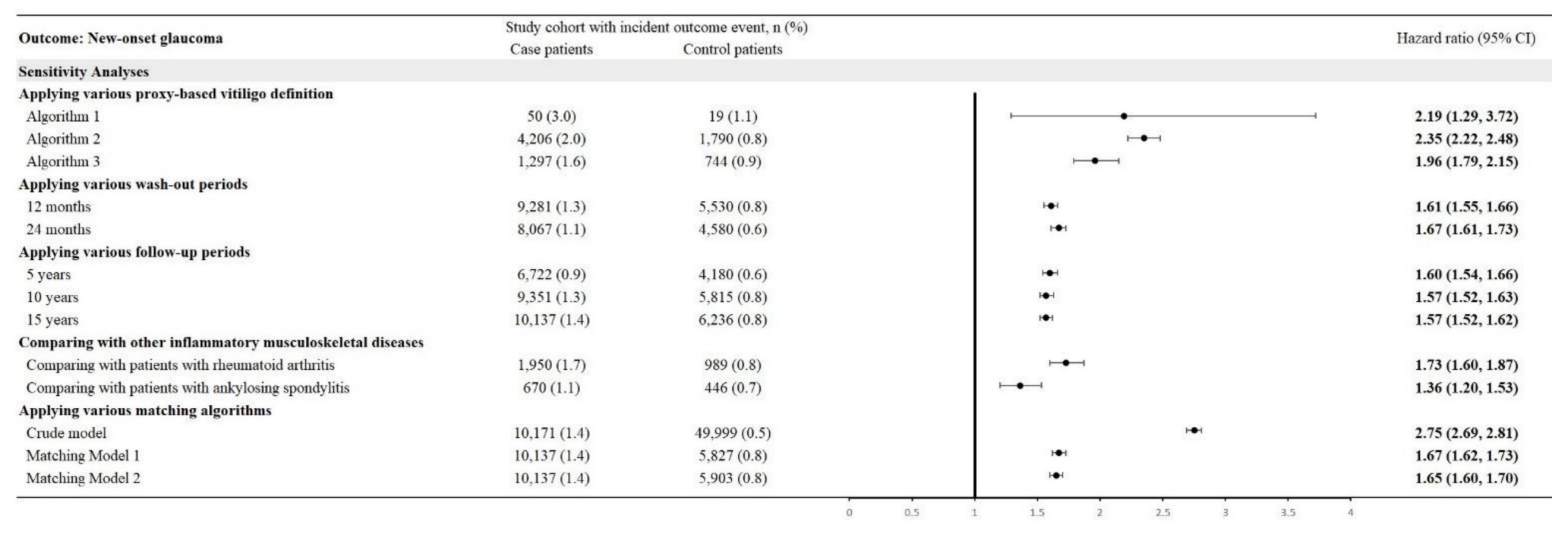
Sensitivity analysis using various models.

Using three proxy-based definitions of glaucoma yielded HRs of 2.19 (95% CI: 1.29-3.72) for Algorithm 1, 2.35 (95% CI: 2.22-2.48) for Algorithm 2, and 1.96 (95% CI: 1.79-2.15) for Algorithm 3. With washout periods of 12 and 24 months, HRs were 1.61 (95% CI: 1.55-1.66) and 1.67 (95% CI: 1.61-1.73), respectively. Follow-up periods of 5, 10, and 15 years produced HRs of 1.60 (95% CI: 1.54-1.66), 1.57 (95% CI: 1.52-1.63), and 1.57 (95% CI: 1.52-1.62), respectively. Compared with other inflammatory musculoskeletal diseases, CTS patients had an HR of 1.73 (95% CI: 1.60-1.87) versus rheumatoid arthritis and 1.36 (95% CI: 1.20-1.53) versus ankylosing spondylitis. Crude and matched models yielded HRs of 2.75 (95% CI: 2.69-2.81), 1.67 (95% CI: 1.62-1.73), and 1.65 (95% CI: 1.60-1.70) (**Figure 4**).

### Stratification Analysis

**Figure 5:**
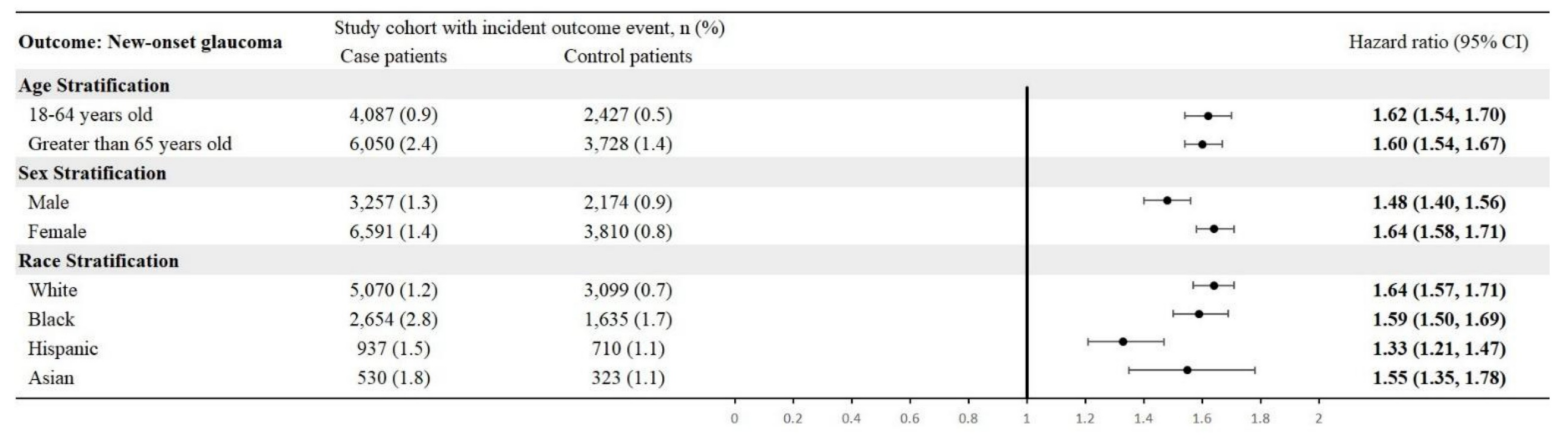
Stratified analysis by age, sex, and race.

By age, CTS patients aged 18-64 had an HR of 1.62 (95% CI: 1.54-1.70), and those > 65 had an HR of 1.60 (95% CI: 1.54-1.67). In sex-specific analyses, males showed an HR of 1.48 (95% CI: 1.40-1.56) and females 1.64 (95% CI: 1.58-1.71). Race-stratified HRs were 1.64 (95% CI: 1.57-1.71) for White, 1.59 (95% CI: 1.50-1.69) for Black, 1.33 (95% CI: 1.21-1.47) for Hispanic, and 1.55 (95% CI: 1.35-1.78) for Asian patients (**Figure 5**).

## Discussion

In this large-scale retrospective cohort study, we found that people with carpal tunnel syndrome are associated with a significantly increased risk of future glaucoma. Patients with CTS had approximately a 57% higher hazard of developing glaucoma than comparable individuals without CTS (HR 1.57, 95% CI 1.52-1.62). This risk elevation was evident after rigorous matching for age, sex, race, and key comorbidities, suggesting an independent association between CTS and glaucoma.

Potential mechanisms linking CTS and glaucoma may be attributed to shared pathophysiological pathways, particularly inflammation and immune dysregulation. CTS is not solely a result of mechanical compression but also includes an inflammatory component^14^. Chronic median nerve compression induces microvascular injury and inflammatory changes, with evidence of synovial tissue proliferation and inflammatory cell infiltration within the carpal tunnel^15^. Pro-inflammatory cytokines, such as TNF-α and IL-6, have been detected in higher concentrations in the tenosynovium of CTS patients, especially in severe or long-standing cases, suggesting that inflammation contributes to the pathogenesis and progression of median nerve injury^16^, ^17^. Similarly, glaucoma is increasingly recognized as an ocular neuroinflammatory disease. In primary open-angle glaucoma, where elevated intraocular pressure is a known risk factor, studies have shown activation of glial cells in the optic nerve head and retina^18^, releasing inflammatory mediators that may exacerbate neurodegeneration. We suppose that such a process could involve the attack on synovial tissues in the wrist, leading to or exacerbating CTS while also contributing to optic nerve damage or impaired ocular fluid dynamics, which may facilitate glaucoma. These observations suggest that an individual's pro-inflammatory state or autoimmune environment could be a key factor linking the two conditions.

In addition to the common inflammation patterns, neurodegenerative vulnerability and vascular factors provide further insight into the association between CTS and glaucoma. Both conditions have the involvement of neuropathies. CTS involves the peripheral nervous system, while glaucoma affects the optic nerve, anatomically part of the central nervous system^2^, ^6^. Evidence suggests that neurodegeneration in glaucoma extends beyond the eye, with imaging and functional studies demonstrating alterations in the lateral geniculate nucleus and visual cortex^19^. Some glaucoma patients also present with changes in the peripheral nervous system, including signs of autonomic dysfunction^20^. Although the pathophysiology of CTS is primarily mechanical due to nerve compression, the severity of nerve injury may be modulated by systemic factors such as ischemic tolerance, oxidative stress, and the ability to repair axonal damage^21^, ^22^, as these factors could potentially contribute to optic nerve vulnerability in glaucoma. From a vascular perspective, both CTS and glaucoma may be exacerbated by impaired microcirculation. In CTS, elevated pressure within the carpal tunnel compromises epineural blood flow to the median nerve, leading to localized ischemia and endoneurial edema^10^. In glaucoma, particularly normal-tension glaucoma, vascular insufficiency is strongly implicated^23^. Inadequate perfusion of the optic nerve head due to factors such as arterial stiffness, systemic hypotension, or vasospasm^24^ could increase the vulnerability of retinal ganglion cells, even in the presence of normal intraocular pressure. However, the precise mechanisms underlying the observed association between CTS and glaucoma remain unclear and warrant further investigation in future studies.

However, the association observed between CTS and angle-closure glaucoma warrants cautious interpretation, given the predominantly anatomical nature of angle-closure disease. Angle-closure glaucoma is primarily determined by structural factors such as lens position, and iridocorneal angle configuration 25, which are distinct from the neuroinflammatory and microvascular pathways more commonly implicated in open-angle glaucoma. Therefore, it is unlikely that CTS directly contributes to angle closure through a shared inflammatory or neuropathic mechanism, and the observed association with angle-closure glaucoma may reflect shared risk profiles or detection effects rather than a direct causal pathway. These considerations underscore the importance of interpreting subtype-specific associations within the context of distinct glaucoma pathophysiology.

The retrospective cohort design of this study does not allow conclusions regarding causality between CTS and glaucoma. Although algorithms requiring repeated encounters and supplementary codes were applied to improve diagnostic accuracy, both CTS and glaucoma definitions relied on administrative coding rather than standardized clinical confirmation. Moreover, the global nature of the TriNetX network means that diagnostic criteria and coding practices may vary across participating sites. Such heterogeneity could influence disease ascertainment and contribute to variability in the observed associations. These issues highlight the need for future prospective studies integrating standardized ophthalmologic and neurologic assessments to validate our findings.

Importantly, our glaucoma subtype analyses provide indirect evidence that the observed association is unlikely to be driven primarily by corticosteroid exposure. Corticosteroid use is a well-recognized cause of secondary glaucoma and represents a plausible confounder in patients with CTS, who may receive local or systemic steroids as part of conservative management 26. However, in our study, CTS was significantly associated with primary glaucoma, whereas the association with secondary glaucoma was not statistically significant (**Figure 3**). This divergence suggests that steroid-induced ocular hypertension alone does not explain the increased glaucoma risk observed in CTS patients. Instead, the predominance of primary glaucoma supports the hypothesis that shared pathophysiological mechanisms such as chronic inflammation, neurodegenerative susceptibility, or microvascular dysfunction may underlie both CTS and glaucomatous optic neuropathy.

This study possesses several strengths that enhance the validity of its findings. Chief among them is the applied large-scale dataset in the current study, derived from the TriNetX network, which includes patient information from 147 healthcare organizations across North America, Europe, and Asia. The cohort comprises over 700,000 individuals with CTS and an equally large matched control group. This broad, multicenter representation improves the generalizability of the results. Furthermore, the substantial sample size provides high statistical power to detect moderate associations and enables detailed subgroup analyses by glaucoma subtype and demographic characteristics. Another strength is the variability of controls applied in the current study. Although the primary comparison was made between patients with carpal tunnel syndrome and a general health check-up cohort, additional analyses were conducted using alternative comparator groups. CTS patients were compared to individuals with other autoimmune orthopedic conditions (rheumatoid arthritis and ankylosing spondylitis) closely associated with glaucoma. The consistent elevation in risk observed across these comparator groups supports the robustness of the findings. It reduces the likelihood that selection bias in the control group choice substantially influenced the results.

However, several limitations should be acknowledged. First, this was a retrospective analysis of electronic health record data, which inherently limits the ability to establish causality. Diagnoses of CTS and glaucoma were identified using ICD-10-CM and administrative codes, rather than standardized clinical confirmation, and therefore may be subject to inaccuracies. Although we applied algorithms requiring repeated encounters and, in sensitivity analyses, incorporated adjunctive treatments or procedures to strengthen case validity, misclassification cannot be fully excluded. Mild or subclinical cases may also have been underreported. In addition, in our definitions, Q15.0 (congenital glaucoma) was included under the primary glaucoma category to capture potential cases of primary congenital glaucoma, which lacks a specific ICD-10-CM code. However, this decision may have introduced further misclassification, as Q15.0 can also encompass broader congenital anomalies, potentially diluting or exaggerating the observed associations. Moreover, because TriNetX integrates data from multiple international health systems, diagnostic criteria and coding practices may vary across countries and institutions. Such heterogeneity could influence disease ascertainment and introduce variability into the results. Second, despite extensive propensity score matching, certain clinically relevant confounders were not captured in the TriNetX database. These include family history of glaucoma, detailed ophthalmologic examination data (such as intraocular pressure levels, optic nerve head imaging, and visual field results), occupational and ergonomic risk factors related to CTS, and detailed information on concomitant medication use, particularly dosage of systemic or ophthalmic corticosteroid exposure. The lack of these variables introduces the possibility of residual confounding, as each of them represents established or plausible risk factors for glaucoma. For instance, corticosteroid therapy has long been recognized as a contributor to secondary glaucoma, while family history remains one of the strongest predictors of primary glaucoma. Similarly, visual strain associated with occupational exposures may increase the likelihood of ophthalmologic consultations and subsequent diagnosis. Without the ability to control for these factors, the hazard ratio estimates reported in this study should be interpreted with caution. Third, a surveillance effect may have contributed to the observed association, as individuals with CTS often undergo more frequent clinical evaluations than controls, increasing the chance of glaucoma being detected. In the sensitivity analysis, we attempted to address this by comparing CTS patients with those diagnosed with other inflammatory musculoskeletal conditions. Nevertheless, as we could not directly adjust for the frequency of eye examinations, the potential impact of surveillance bias should be cautiously considered. Fourth, although TriNetX contains data from more than 144 million patients worldwide, the study population reflects individuals actively engaged with healthcare systems rather than community-based cohorts. As such, the findings may not be entirely generalizable to populations with limited healthcare access or to those who do not routinely seek medical care. In addition, because the data are collected from specific participating health systems, the cohorts may not fully mirror the broader global population. Regional differences in healthcare availability, diagnostic practices, coding intensity, and follow-up intensity may contribute to heterogeneity in case ascertainment and influence risk estimates. Furthermore, since TriNetX integrates heterogeneous electronic health record systems, the completeness and accuracy of documentation may vary across institutions. Missingness in important covariates, such as BMI and socioeconomic status, may further compromise matching quality and outcome ascertainment, introducing potential bias. These issues should be considered when evaluating the external validity of our findings and their applicability beyond the populations captured in TriNetX. Fifth, given that our cohort spanned more than 10 years, improvements in diagnostic technology, coding systems, and clinical practices may have influenced the recognition of glaucoma over time. Such temporal heterogeneity could differentially affect CTS and non-CTS groups and may have introduced bias.

In conclusion, in this large-scale, global, real-world cohort study, we report that patients with CTS had a significantly increased risk of developing glaucoma, and the association remained robust across demographic groups, glaucoma stages, and multiple sensitivity analyses. These findings suggested that CTS may be a clinical marker for heightened glaucoma susceptibility. Early ophthalmologic evaluation in patients with CTS could help facilitate the timely detection and management of glaucoma, highlighting the importance of interdisciplinary vigilance in clinical care.

## Supplementary Material

Supplementary tables.

## Figures and Tables

**Table 1:** Baseline characteristics

	Before matching				After matching^a^		
	CTS cohort (n = 736,975)	CTS-free cohort (n = 9,546,008)	SMD		CTS cohort (n = 733,997)	CTS-free cohort (n = 733,997)	SMD
**Age at index**							
Mean±SD	50.9 ± 15.7	38.0 ± 20.1	0.71		50.9 ± 15.7	50.9 ± 15.7	0.00
**Sex**							
Male	247126 (33.7)	4128380 (43.4)	0.20		247126 (33.7)	246524 (33.6)	0.00
Female	464455 (63.3)	5022893 (52.9)	0.21		464455 (63.3)	464436 (63.3)	0.00
Unknown Gender	138818 (18.9)	2053952 (21.6)	0.07		138818 (18.9)	134703 (18.4)	0.01
**Race, n (%)**							
White	435283 (59.3)	5201253 (54.7)	0.09		435283 (59.3)	435307 (59.3)	0.00
Black or African American	95928 (13.1)	1234441 (13.0)	0.00		95928 (13.1)	93407 (12.7)	0.01
Asian	29137 (4.0)	462526 (4.9)	0.04		29137 (4.0)	33990 (4.6)	0.03
American Indian or Alaska Native	3212 (0.4)	33948 (0.4)	0.01		3212 (0.4)	2460 (0.3)	0.02
Native Hawaiian or Other Pacific Islander	5581 (0.8)	49419 (0.5)	0.03		5581 (0.8)	3615 (0.5)	0.03
Other Race	26038 (3.5)	468457 (4.9)	0.07		26038 (3.5)	30515 (4.2)	0.03
Unknown Race	22416 (3.1)	352723 (3.7)	0.04		22416 (3.1)	23037 (3.1)	0.00
**Socioeconomic status**							
Persons with potential health hazards related to socioeconomic and psychosocial circumstances	14016 (1.9)	117827 (1.2)	0.05		14016 (1.9)	14015 (1.9)	0.00
**Lifestyle**							
Mental and behavioral disorders due to psychoactive substance use	87281 (11.9)	482719 (5.1)	0.25		87281 (11.9)	87324 (11.9)	0.00
**Medical Utilization**							
Visit: Ambulatory	572069 (77.9)	6007902 (63.2)	0.33		572069 (77.9)	537346 (73.2)	0.11
Visit: Inpatient Encounter	179149 (24.4)	1335692 (14.1)	0.26		179149 (24.4)	179153 (24.4)	0.00
**BMI**							
Greater than 25 kg/m^2^	296357 (40.4)	2116930 (22.3)	0.40		296357 (40.4)	296443 (40.4)	0.00
**Comorbidities**							
Essential hypertension	176221 (24.0)	1126231 (11.9)	0.32		176221 (24.0)	176192 (24.0)	0.00
Hyperlipidemia	106184 (14.5)	639090 (6.7)	0.25		106184 (14.5)	95746 (13.0)	0.04
Diabetes mellitus	81369 (11.1)	441439 (4.6)	0.24		81369 (11.1)	66043 (9.0)	0.07
Chronic ischemic heart disease	34483 (4.7)	207735 (2.2)	0.14		34483 (4.7)	32515 (4.4)	0.01
Chronic kidney disease	18453 (2.5)	128490 (1.4)	0.08		18453 (2.5)	19532 (2.7)	0.01
Psoriasis	7371 (1.0)	42438 (0.4)	0.07		7371 (1.0)	5445 (0.7)	0.03
Vitamin D deficiency	56489 (7.7)	314421 (3.3)	0.19		56489 (7.7)	45601 (6.2)	0.06
Ankylosing spondylitis	1178 (0.2)	5254 (0.1)	0.03		1178 (0.2)	684 (0.1)	0.02
Rheumatoid arthritis	3430 (0.5)	11766 (0.1)	0.06		3430 (0.5)	3077 (0.4)	0.01

CTS, Carpal tunnel syndrome; SMD, standardized mean difference; SD, Standardized difference. ^a^ Propensity score matching was performed in a 1:1 ratio based on the following variables: age at index date, sex, race, body mass index (BMI), medical utilization (inpatient encounters), lifestyle factors (mental and behavioral disorders related to psychoactive substance use), socioeconomic status (individuals exposed to potential health risks due to socioeconomic and psychosocial conditions), and comorbidities (rheumatoid arthritis and hypertension).
