# Supplementary material for: Risk of new-onset glaucoma in people with carpal tunnel syndrome: a global-federated, multicenter retrospective cohort study
Source: Int J Med Sci. 2026 Jan 14;23(2):661–9. doi: 10.7150/ijms.122874 (PMC12825145; doi:10.7150/ijms.122874)
Supplement: Supplementary file 1 — Supplementary tables. [file ijmsv23p0661s1.pdf]

Supplementary File

Table S1. Utilized proxy codes [a]

**Description**	**ICD-10-CM codes**
Study population	
Carpal tunnel syndrome	ICD-10-CM G56.0
Outcome events	
Glaucoma or glaucoma-related diagnoses	ICD-10-CM: H40-H42
Primary glaucoma	ICD-10-CM: H40.11, H40.2, Q15.0
Secondary glaucoma	ICD-10-CM: H40.3-H40.6
Open-angle glaucoma	ICD-10-CM: H40.1
Angle-closure glaucoma	ICD-10-CM: H40.2
Glaucoma suspect	ICD-10-CM: H40.0
Preglaucoma	ICD-10-CM: H40.00
Open angle with borderline findings	ICD-10-CM: H40.01-H40.02
Anatomical narrow angle	ICD-10-CM: H40.03
Ocular hypertension	ICD-10-CM: H40.05
Covariates and other definitions	
Essential hypertension	ICD-10-CM: I10
Hyperlipidemia	ICD-10-CM: E78.5
Diabetes mellitus	ICD-10-CM: E08-E15
Chronic ischemic heart disease	ICD-10-CM: I25
Chronic kidney disease	ICD-10-CM: N18

Psoriasis	ICD-10-CM: L40
Vitamin D deficiency	ICD-10-CM: E55
Rheumatoid arthritis	ICD-10-CM: M05
Ankylosing spondylitis	ICD-10-CM: M45
Persons with potential health hazards related to socioeconomic and psychosocial circumstances	ICD-10-CM: Z55-Z65
Mental and behavioral disorders due to psychoactive substance use	ICD-10-CM: F10-F19
Encounter for general examination	ICD-10-CM: Z00

[a]ICD-10-CM: International Classification of Diseases, Tenth Revision, Clinical Modification

Table S2. Description of all applied sensitivity analysis models

Models	Description
**Applying various proxy-based CTS definition**	
Algorithm 1	Only patients with documented encounters with the ICD-10-CM diagnosis code G56.0 (Carpal tunnel syndrome) with the application of splint (HCPCS code: A4570) were classified into the CTS cohort for this model.
Algorithm 2	Patients were included in the CTS cohort for this model only if they had a documented diagnosis of Carpal tunnel syndrome (ICD-10-CM code G56.0) along with a prescription record for Non-Steroidal Anti-Inflammatory Analgesics (VA code: CN104).
Algorithm 3	Patients were included in the CTS cohort for this model only if they had a documented diagnosis of CTS (ICD-10-CM code G56.0) along with record for median nerve releasing surgery (CPT code: 64721).
**Applying different wash-out period after index date**	
12 months/24 months after index date	Any outcome events that took place during the specified washout period were not included in the analyses that followed. A consistent 15-year follow-up timeframe was used across all sub-analyses.
5 years /10 years /15 years after index date	Only outcome events that occurred within the established 15-year follow-up window were included in the analysis. A uniform three-month washout period was implemented in each sub-analysis.
**Applying different matching covariates**	
Crude model	Hazard ratio was calculated based on the population without propensity score matching
Matching model 1	Matching covariates include age at index and sex

Matching model 2	Matching covariates include age at index, sex, socioeconomic status, lifestyle (mental and behavioral disorders due to psychoactive substance use)
**Applying various active comparators**	
Comparing with rheumatoid arthritis patients/ Comparing with ankylosing spondylitis patients	In each sub-analysis, the control group will include the corresponding inflammatory musculoskeletal conditions that have been previously identified as being associated with an elevated risk of developing new-onset glaucoma. All sub-analyses will use a follow-up period of 15 years and a wash-out period of 3 months.
